# Three cases of sporadic meningioangiomatosis with different imaging appearances: case report and review of the literature

**DOI:** 10.1186/s12957-015-0477-x

**Published:** 2015-02-28

**Authors:** Zhihua Sun, Fei Jin, Jing Zhang, Yue Fu, Wei Li, Hong Guo, Yunting Zhang

**Affiliations:** Department of Radiology, Tianjin Medical University General Hospital, No. 154, Anshan Dao Road, Heping District, Tianjin, 300052 People’s Republic of China

**Keywords:** Meningioangiomatosis, Magnetic resonance imaging, Flow void effect, Cystic meningioangiomatosis

## Abstract

**Background:**

Meningioangiomatosis (MA) is a rare meningiovascular malformation or hamartomatous lesion in the central nervous system. Radiographic findings of MA may show a variety of characteristics according to different histological components. We present three cases of sporadic MA with different imaging appearances in an attempt to identify specific imaging characteristics.

**Case presentation:**

In case 1, an irregular hyperdense solid mass was localized in the left middle cranial fossa, demonstrating low and equal signal intensity on T1-weighted imaging (T1WI; TR/TE 2,048.9 ms/26.1 ms), high signal intensity with multiple flow void effect on T2-weighted imaging (T2WI; TR/TE 4,000 ms/106.4 ms), and significant and homogeneous enhancement on post-contrast magnetic resonance imaging (MRI). In case 2, the lesion in the right insular lobe showed a cystic-mural nodule pattern. The cystic content demonstrated similar density or signal intensity as cerebrospinal fluid, while the mural nodule demonstrated equal density or signal intensity on computed tomography (CT) and MRI. On post-contrast MRI, the mural nodule showed significant enhancement, but the cystic wall and content showed no enhancement. In case 3, a remarkably enhanced solid nodule was found in the cortex of the left parietal lobe with multiple small cysts surrounding it. This nodule showed low signal intensity on T2WI and diffusion-weighted imaging (DWI; TR/TE 6,000 ms/96.8 ms, *b* = 1,000 s/mm^2^). The preoperative diagnoses of the above three cases were meningioma, hemangioblastoma, and ganglioglioma. However, all were pathologically diagnosed as MA.

**Conclusion:**

The presented cases demonstrate that MA may present with solid and cystic imaging patterns, which may include large cystic-mural nodules and small intra- and extra-cystic patterns. Although MA imaging diagnoses are difficult, several MRI signs may include specific characteristics, such as a flow void effect on T2WI and separating cysts in the cystic MA (as shown in our cases), gyriform hyperintensity on T2-fluid attenuated inversion recovery (FLAIR) sequence, and susceptibility artifacts on T2 gradient echo (GRE) sequences (as found in the literature).

## Background

Meningioangiomatosis (MA) is a rare meningiovascular malformation or hamartomatous lesion in the central nervous system, which was first described by Bassoe and Nuzum [1] and then was named by Worster-Drought *et al*. [2]. MA may occur sporadically or in association with neurofibromatosis (NF) type 2. The pathological characteristics of MA include leptomeningeal calcification and meningovascular proliferation interwoven with bands of fibrous connective tissue. The radiographic findings of MA may demonstrate a variety of characteristics according to different histological components. In this study, we present three cases of sporadic MA with different imaging appearances and attempt to identify specific characteristics to help in preoperative diagnoses. In addition, the relevant medical literature was reviewed.

## Case presentation

### Case 1

**Figure 1:**
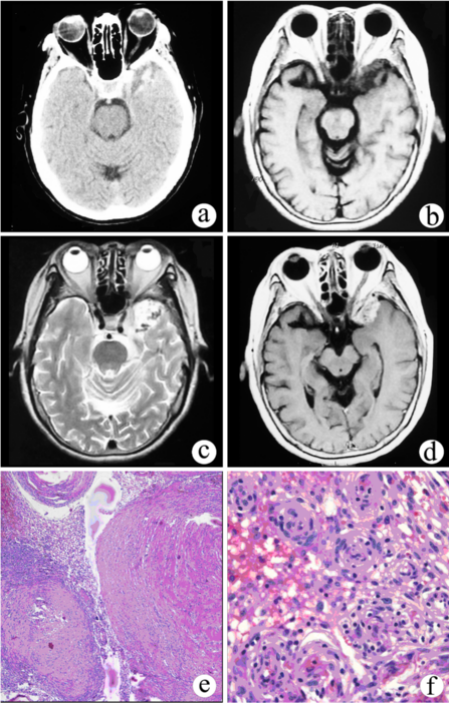
**Solid meningioangiomatosis. (a)** CT scan showed an irregular mixed high-density mass in the left middle cranial fossa. **(b)** On T1WI, the lesion demonstrated low and equal signal intensity. **(c)** On T2WI, the lesion showed high signal intensity with a multiple flow void effect. **(d)** On post-contrast MRI, the lesion showed significant and homogeneous enhancement. **(e**, **f)** Microphotography of specimens showed extensive fibroblastic proliferation and an increased number of vessels surrounded by meningothelial cells.

A 73-year-old female patient had a history of binocular diplopia for 1 week. Physical examination showed a limitation in abduction movement in the left eye. A head computed tomography (CT) scan showed an irregular mixed hyperdense mass in the left middle cranial fossa (Figure 1a). On magnetic resonance imaging (MRI), the lesion demonstrated low and equal signal intensity on T1-weighted imaging (T1WI; TR/TE 2,048.9 ms/26.1 ms) and high signal intensity with multiple flow void effect on T2-weighted imaging (T2WI; TR/TE 4,000 ms/106.4 ms) (Figure 1b,c). On post-contrast MRI, the lesion showed significant and homogeneous enhancement after gadolinium diethylenetriamine pentaacetate (Gd-DTPA) was administered. The margin between the lesion and the adjacent brain cortex was well demarcated, and there was no obvious mass effect. The preoperative diagnosis was meningioma.

The patient underwent a left temporal craniotomy, and complete removal of the tumor was performed. The tumor was firm, pink, and enveloped and had a general blood supply. The base was located in the side wall of the cavernous sinus and sphenoid crest. Microscopically, there was extensive fibroblastic proliferation and an increased number of vessels surrounded by meningothelial cells (Figure 1e,f). Pathological diagnosis was MA.

### Case 2

**Figure 2:**
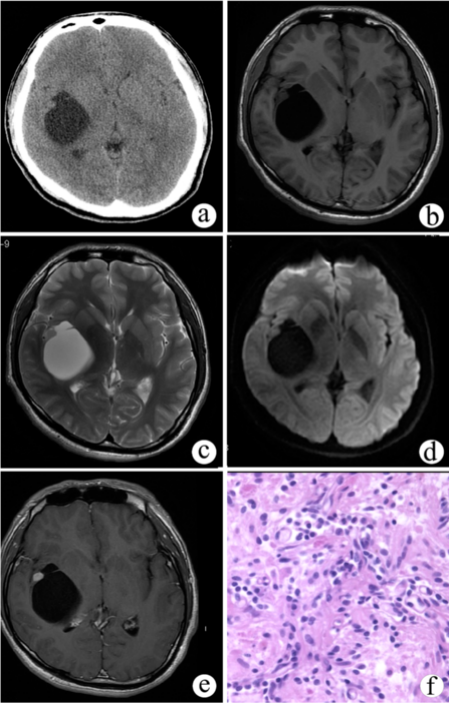
**Cystic meningioangiomatosis with a cystic-mural nodule pattern. (a)** CT scan showed a cystic-mural nodule lesion in the right insular lobe. **(b-d)** In non-enhanced MRI, the cystic content demonstrated similar signal intensity as cerebrospinal fluid (CSF), while the mural nodule demonstrated iso-signal intensity on T1WI, T2WI, and DWI. **(e)** On post-contrast MRI, the mural nodule demonstrated significant enhancement, while the cystic wall and content showed no enhancement. **(f)** Pathological examination showed perivascular spindle-cell proliferation.

A 23-year-old male patient presented with left hemianesthesia for more than 2 years. Physical examination showed hypesthesia and slight ataxia on the left side, and a decrease of graphics and two-point discrimination sensation. A cystic-mural nodule lesion was localized in the right insular lobe on head plain CT (Figure 2a). The cystic portion had low density with 9 Hu, and the mural nodule was isodense with 35 Hu. The cystic content demonstrated similar signal intensity as cerebrospinal fluid (CSF), while the mural nodule demonstrated iso-signal intensity on T1WI, T2WI, and diffusion-weighted imaging (DWI; TR/TE 6,000 ms/96.8 ms, *b* = 1,000 s/mm^2^) (Figure 2b,c,d). On post-contrast MRI, the mural nodule showed significant enhancement, but the cystic wall and content demonstrated no enhancement (Figure 2e). The adjacent brain parenchyma and sulci were compressed and deformed, but there was no edema around the tumor. The preoperative diagnosis was hemangioblastoma.

The patient underwent a right temporal craniotomy. Upon operation, approximately 50 ml of a yellow transparent cyst fluid was extracted from the cystic tumor. The purple mural nodule was approximately 8 mm in diameter with fibrous adhesion to the right insular cortex. Pathological examination demonstrated a classic MA appearance (Figure 2f).

### Case 3

**Figure 3:**
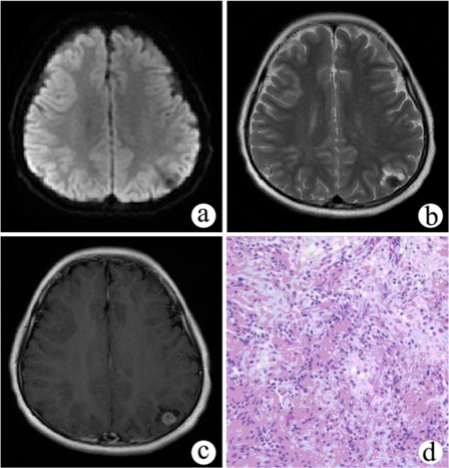
**Cystic meningioangiomatosis with a multiple microcystic pattern. (a, b)** DWI and T2WI demonstrated a low signal intensity nodule in the left parietal cortex and multiple small cysts surrounding it. **(c)** On post-contrast MRI, the nodule was remarkably enhanced. **(d)** Microscopically, fibroblast-like spindle cells were arranged in a spiral shape around multiple vessels, and the cortical neurons were entrapped within the lesion.

A 9-year-old girl suffered from an involuntary convulsion in her right limb for 1 month. Physical examination showed no positive signs. On non-enhanced MRI, a solid nodule 11 mm in diameter was found in the cortex of the left parietal lobe, demonstrating low signal intensity on DWI and T2WI (Figure 3a,b). Multiple small cysts were around the nodule. There was a slight mass effect and surrounding edema. After Gd-DTPA administration, the nodule was remarkably enhanced (Figure 3c). The preoperative diagnosis was ganglioglioma.

The patient underwent a left parietal craniotomy, and the tumor was completely removed. The tumor was purple with an abundant blood supply, and it was unclearly demarcated from adjacent brain tissue. Microscopically, fibroblast-like spindle cells were arranged in spiral shape around multiple vessels, and cortical neurons were entrapped within the lesion (Figure 3d). The pathological diagnosis was MA.

### Discussion

MA can be divided into two categories: sporadic or associated with NF type 2. Sporadic meningioangiomatosis usually occurs in young adults or children who present with seizures or headaches. In contrast, NF2-associated cases are usually asymptomatic and discovered post-mortem. MA has a slight male predominance [3], but according to some authors [4], it has no sex predilection. Meningioma is the most commonly associated neoplasm, and other associated abnormalities include cerebral hemorrhage, oligodendroglioma, arteriovenous malformations, cerebral softening, and meningeal hemangiopericytoma [5].

MA was mostly localized in the supratentorial region, particularly in the temporal or frontal lobes. Sporadic MA has mainly been reported in the temporal lobe (33%) followed by the frontal lobe (25%), but MA accompanied by NF has been shown to mostly occur in the frontal lobe (35%) [6,7]. Atypical locations have also been reported [8], including the third ventricle, thalamus, cerebral peduncle, and brain stem. Multiple MA lesions accompanied by NF have occurred in 35% of patients, but the incidence of sporadic MA was only 13% [9].

The pathogenesis of MA remains unclear. Proposed hypotheses include hamartoma with degenerative changes, leptomeningeal meningioma with invasion in adjacent brain tissue, and cortical vascular malformation. MA was formerly classified as a neurocutaneous syndrome, and some cases associated with NF supported the theory of a hamartoma [10]. Although not all cases have a meningeal component and malignancy characteristics are typically absent, the association between meningioma in rare cases and molecular aberrations regarding the NF2 gene in both lesions suggests that MA may be correlated with meningioma. Kim *et al*. [11] found that the meningiomatosis portions of meningiomatosis-meningioma have loss of heterozygosity for the 22q12 locus in 28.6% (2/7) of coexisting cases of meningiomatosis-meningioma, whereas each pure meningiomatosis harbors one loss of heterozygosity at either 22q12 or 9p21. The theory of cortical vascular malformation corresponded to the histopathological characteristic of MA. Cortical vascular malformation induced the perivascular meningothelial proliferation of cells from vessel walls or pluripotent arachnoid cap cells in Virchow-Robin spaces. Leptomeninges and arachnoid cap cells normally surround blood vessels as they penetrate the cortex. Conceivably, chronic leptomeningeal stimulation by underlying cortical lesions could result in MA histopathological changes.

Histopathologically, MA has been regarded as a hamartomatous, reactive, or neoplasic lesion originating from multivariate cells, such as meningothelial, fibroblastic, myofibroblastic, smooth muscle, or pluripotent cells [12]. The characteristics of MA include leptomeningeal proliferation accompanied by calcification or psammoma body formation, perivascular cuffs of spindle-shaped fibroblast-like cells, whorls, or bands of meningothelial cells in association with sharply demarcated intracortical plaques of proliferating small vessels. In our three cases, all had the specific histological characteristics mentioned above in the solid portion, but calcification was found only in case 1. Other histological abnormalities included gliosis, neuronal dysplasia, cystic degeneration of white matter, and vascular hyalinization.

Immunohistochemical staining of MA was relatively unhelpful for diagnosis. Positive vimentin expression reflected the proliferation of blood vessels and perivascular fibroblast-like cells in almost all cases. However, the results of other immunohistochemical markers, such as epithelial membrane antigen (EMA), S-100 protein, and glial fibrillary acidic protein (GFAP), were variable in the literature [10].

According to different percentages of perivascular cell proliferation and cortical vascular proliferation, MA may be broadly classified into predominantly cellular or vascular types [13]. Predominantly cellular MA has moderate to high cellularity such as that found in cases 2 and 3 (Figures 2f and 3d). In the central portion of the lesion, MA cells emerge from the perivascular location and infiltrate the cortex. Approximately 90% of reported cases have cortical invasion, but the proliferating cells have no significant atypia, mitoses, or necrosis. Predominantly vascular MA contains thick-walled, hyalinized, and calcified blood vessels with minimal perivascular cell proliferation, which is observed in case 1 (Figure 1e,f). Hemorrhage was commonly found in this subtype.

It has been observed that there are no specific MA characteristics upon MRI or CT; thus, correct preoperative MA diagnoses were difficult. MA has always been misdiagnosed as meningioma, oligodendroglioma, ganglioglioma, metastasis, or arteriovenous malformation. With regards to the location of lesions, they may manifest as intra-axial or extra-axial lesions with well- or ill-defined margins because of the cortex invasion. CT scans show that lesions exhibit different densities or even normal appearance, but calcification may be a specific indication for MA. The probability of calcification is variable, and the highest observed was 89.6% [14]. The signal intensity in MR images was iso- to hypointense on T1-weighted images and hypo- to hyperintense on T2-weighted images. In case 1, multiple flow void effect could be observed within MA on T2WI. To our knowledge, this characteristic was first described for MA. This effect corresponded to a predominantly vascular histological type, that is, proliferating vessels. Contrast-enhanced images also showed various types of enhancement patterns ranging from mild to strong and even no enhancement, but remarkable enhancement was the most common pattern with a prevalence of 79.6% [14]. The mass effect was none or slight. Surrounding edema was always absent, but adjacent subcortical white matter was occasionally hyperintense on T2WI resulting from edema or gliosis [15].

Some specific signs on MRI may be helpful for MA imaging diagnoses. Gyriform hyperintense on T2-fluid attenuated inversion recovery (FLAIR) sequence has been reported to be the most prominent characteristic for sporadic MA on MR imaging, which can be attributed to a thickened cortex with proliferating leptomeningeal vessels interwoven with bands of fibrous connective tissue [13]. Calcification may be ignored on routine T1WI and T2WI, but it produces a susceptibility artifact on T2 gradient echo (GRE) sequences [14]. Chronic hemosiderin from cavernous malformations also showed susceptibility artifacts; thus, T2 GRE sequences may only be thought of as a reference imaging modality for MA diagnoses. Magnetic resonance spectrum (MRS) can aid in the evaluation of brain lesions by analyzing the spectrum of metabolites present in the area of study. Rokes *et al*. [16] have reported a case of MA with a distinct choline (Cho) peak and a decreasing *N*-acetyl aspartate (NAA) peak on MRS, which suggested a proliferating tumor of presumed non-neuronal origin. The high Cho peak could have been a reflection of the proliferation of meningothelial cells and/or fibroblasts that formed the concentric cuffs surrounding blood vessels in the cortex. However, the high Cho peak could have also been observed under other conditions, for example, high-grade brain tumors [17].

We presumed that the MA imaging appearances could be divided into two patterns: solid and cystic. Solid MA was more common than cystic MA and demonstrated a higher probability of calcification and remarkable enhancement in most cases. Less than ten cases with cystic MA have been reported [18-23], including cases 2 and 3, although the total number of reported MA cases is approximately 120. Cysts were localized within or around solid tumors, one or multiple, and demonstrated micro- or macrocysts. The exact mechanism underlying MA cyst development remains controversial. Park *et al*. [22] speculated that cysts were due to the accumulation of CSF within lesions in a manner similar to the mechanism of cyst formation in cystic meningiomas. Supported evidence included cysts that were eccentric to the solid mass and adjacent to sulci. However, communication between cysts and the subarachnoid space has not been confirmed in MA [20]. Another potential mechanism is enlarged perivascular spaces in which CSF gradually accumulates, eventually resulting in the formation of cysts. In our patient, pathologic examination demonstrated that no tumor cells were present in the cyst wall. The cyst was separated from the solid portion of the tumor with no communication between the cyst and subarachnoid space. Therefore, we assumed that enlarged perivascular space may be a major reason for forming cystic MA.

Differential diagnoses for MA are not easy due to variable radiological appearances. Extra-axial tumors, such as meningiomas, are the most common differential diagnosis. Intra-axial tumors in the superficial cortex, such as oligodendroglioma, ganglioglioma, and dysembryoplastic neuroepithelial tumor (DNET), are also frequently considered [24]. However, for cystic MA, we thought that differential tumors should include some cystic-mural tumors with non-enhanced cystic walls, such as hemangioblastomas, pilocystic astrocytomas, and pleomorphic xanthoastrocytomas. The MA cysts are relatively separated from solid masses or nodule, which may be helpful for differential diagnoses. Other non-tumor diseases, that is, arteriovenous malformation, cavernous hemangioma, and granuloma, are occasionally misdiagnosed as MA.

Surgical resection is important not only for seizure control but also for pathological diagnosis. Most MAs are completely removed without recurrence. However, long-term seizures disappeared in only 43% of patients after operation, and antiepileptic drug administration was required in greater than 70% of patients [10,25].

## Conclusions

The cases in this study demonstrated that MA may present with solid or cystic imaging patterns. Imaging diagnoses for MA were difficult due to non-specific characteristics. Several signs on MRI may be helpful, such as gyriform hyperintensity on T2-FLAIR sequences, susceptibility artifacts on T2 GRE sequence, and flow void effects on T2WI as in our case. Cysts in cystic MA demonstrate variable appearances in location, number, and size, but separation from solid masses or nodules may be one of its specific characteristics.

## Consent

Written informed consent was obtained from the patient for the publication of this case report and any accompanying images. A copy of the written consent is available for review by the Editor-in-Chief of this journal.

## Abbreviations

Cho: choline
CSF: cerebrospinal fluid
CT: computed tomography
DNET: dysembryoplastic neuroepithelial tumor
DWI: diffusion-weighted imaging
EMA: epithelial membrane antigen
FLAIR: fluid attenuated inversion recovery
GFAP: glial fibrillary acidic protein
GRE: gradient echo
MA: meningioangiomatosis
MRI: magnetic resonance imaging
MRS: magnetic resonance spectrum
NAA: *N*-acetyl aspartate
NF: neurofibromatosis
T1WI: T1-weighted imaging
T2WI: T2-weighted imaging
